# Urinary Mineral Concentrations in European Pre-Adolescent Children and Their Association with Calcaneal Bone Quantitative Ultrasound Measurements †

**DOI:** 10.3390/ijerph13050471

**Published:** 2016-05-05

**Authors:** Karen Van den Bussche, Diana Herrmann, Stefaan De Henauw, Yiannis A. Kourides, Fabio Lauria, Staffan Marild, Dénes Molnár, Luis A. Moreno, Toomas Veidebaum, Wolfgang Ahrens, Isabelle Sioen

**Affiliations:** 1 Department of Public Health, Ghent University, 4K3, De Pintelaan 185, Ghent 9000, Belgium; Stefaan.DeHenauw@UGent.be (S.D.H.); isabelle.sioen@ugent.be (I.S.); 2 Leibniz Institute for Prevention Research and Epidemiology-BIPS, Achterstraβe 30, Bremen D-28359, Germany; Diana.Herrmann@dguv.de (D.H.); Ahrens@bips.uni-bremen.de (W.A.); 3 Department of Health Sciences, Vesalius, University College Ghent, Keramiekstraat 80, Ghent 9000, Belgium; 4 Child Health Research and Education Institute, 56 Stavrou Street, Strovolos 2035, Cyprus; kourides@cytanet.com.cy; 5 Unit of Epidemiology and Population Genetics, Institute of Food Sciences, Consiglio Nazionale delle Ricerche, Avellino 83100, Italy; fabio.lauria@isa.cnr.it; 6 Department of Pediatrics, Institute of Clinical Sciences, The Queen Silvia Children’s Hospital, Sahlgrenska Academy at University of Gothenburg, Göteborg 416 85, Sweden; staffan.marild@pediat.gu.se; 7 Department of Pediatrics, University of Pécs, József Attila str. 7, Pécs H-7623, Hungary; molnar.denes@pte.hu; 8 GENUD (Growth, Exercise, Nutrition and Development) Research Group, Facultad de Ciencias de la Salud, Instituto Agroalimentario de Aragón (IA2), Instituto de Investigación Sanitaria Aragón (IIS Aragón), Centro de Investigación Biomédica en Red de Fisiopatología de la Obesidad y Nutrición (CIBERObn), Universidad de Zaragoza, C/Domingo Miral s/n, Zaragoza 50009, Spain; lmoreno@unizar.es; 9 Department of Chronic Diseases, National Institute for Health Development, Hiiu str. 42, Tallinn 11619, Estonia; toomas.veidebaum@tai.ee; 10 Institute for Statistics, University of Bremen, Bibliothekestraβe 1, Bremen 28359, Germany; 11 FWO, Research Foundation Flanders, Egmontstraat 5, Brussels 1000, Belgium

**Keywords:** potassium, sodium, calcium, magnesium, phosphate, children, calcaneal quantitative ultrasound

## Abstract

This study investigates differences and associations between urinary mineral concentrations and calcaneal bone measures assessed by quantitative ultrasonography (QUS) in 4322 children (3.1–11.9 years, 50.6% boys) from seven European countries. Urinary mineral concentrations and calcaneal QUS parameters differed significantly across countries. Clustering revealed a lower stiffness index (SI) in children with low and medium urinary mineral concentrations, and a higher SI in children with high urinary mineral concentrations. Urinary sodium (uNa) was positively correlated with urinary calcium (uCa), and was positively associated with broadband ultrasound attenuation and SI after adjustment for age, sex and fat-free mass. Urinary potassium (uK) was negatively correlated with uCa but positively associated with speed of sound after adjustment. No association was found between uCa and QUS parameters after adjustment, but when additionally adjusting for uNa, uCa was negatively associated with SI. Our findings suggest that urinary mineral concentrations are associated with calcaneal QUS parameters and may therefore implicate bone properties. These findings should be confirmed in longitudinal studies that include the food intake and repeated measurement of urinary mineral concentrations to better estimate usual intake and minimize bias.

## 1. Introduction

Reaching an optimal “peak bone mass”, the amount of bone tissue present at the end of the skeletal maturation, has been reported to prevent osteoporosis and associated fractures later in life [1]. Genetic-ethnic factors, body composition, and hormonal status as well as lifestyle behaviour such as physical activity and diet appear to influence peak bone mass [2,3,4,5,6,7,8]. An imbalance in any of these factors can have an impact on bone mass. The main function of bone is to provide mechanical support to protect the internal organs and to act as a repository for the systemic mineral homeostasis [9]. These functions are enabled by the unique composition of bone tissue, the bone matrix containing 35% collagenous organic protein matrix and 65% minerals in the form of hydroxyapatite (HA). HA are crystals of calcium and phosphate (PO_4_, phosphorus (P) bound to oxygen) to which other minerals are conjugated [10]. About 80%–85% of the total PO_4_ in the body, 99% of the total calcium (Ca), 50% to 60% of the total magnesium (Mg) and one-third of total-body sodium (Na) is stored in bone [11,12,13]. The major part of potassium (K) in the body (98%) is present at intracellular level of which a large proportion of the body pool of K is found in muscle and the skeleton [11]. Because bone undergoes continuous remodelling, especially during growth, an adequate intake of micronutrients through nutrition is needed to support bone formation [9,14]. Moreover, it is known that the intake of one mineral can influence the concentration of another mineral in the body. For instance, an excessive intake of Na is considered detrimental for bone as it increases uCa excretion due to an intimate association between renal tubular mechanisms involved in the re-absorption of these ions [15]. A contrasting effect has been reported for the intake of K, which causes a retention of Ca in the tubules [15]. Also Ca and P interact and it is therefore recommended that the Ca/P-ratio in the diet of children must be larger than 1 to enable optimal growth [11]. Dietary intake assessment has been shown to be very difficult in young children, because they are not capable of correctly recalling and estimate their nutritional intake [16]. Measuring urinary mineral concentrations can be an interesting alternative compared to parental-reported intake of these minerals by diet [17]. As it is known that Ca, PO_4_, Mg, Na and K play a role in the bone metabolism, it can be of interest to investigate the association of the urinary concentrations of these minerals with bone health parameters. There are few studies describing this association in children and adolescents. Several studies with varying sample sizes (from 65 to 381 participants) investigated the association between bone health and urinary mineral concentrations such as uK [18], uMg [19], uCa [19,20], urinary potential renal acid load (uPRAL) [21,22] and uNa [18,19,20,21,23] but not uPO_4_. In these studies, bone health was measured via peripheral quantitative computed tomography (pQCT) [21,22] or dual-energy X-ray absorptiometry (DXA) [18,19,20,23].

However, the patterns and role of these urinary mineral concentrations in terms of bone health in healthy young children remains unclear. Therefore, this study aims (1) to describe differences in urinary excretion of uCa, uMg, uPO_4_, uNa and uK in European children aged 3–12 years and (2) to assess the associations between these urinary mineral concentrations and calcaneal bone measures assessed by quantitative ultrasonography (QUS). Therefore, the following hypotheses were tested: 

In healthy pre-school and primary school children:

(1) urinary mineral concentrations differ across countries;
(2) high concentrations of uNa are associated with a higher concentrations of uCa;
(3) high concentrations of uK is associated with a lower concentration of uCa; and
(4) high concentrations of uNa are associated with lower calcaneal QUS parameters.

Due to the availability of a lot of different urinary concentrations, we tested all associations between individual urinary mineral concentrations and QUS parameters.

## 2. Methods

### 2.1. Study Sample

**Figure 1:**
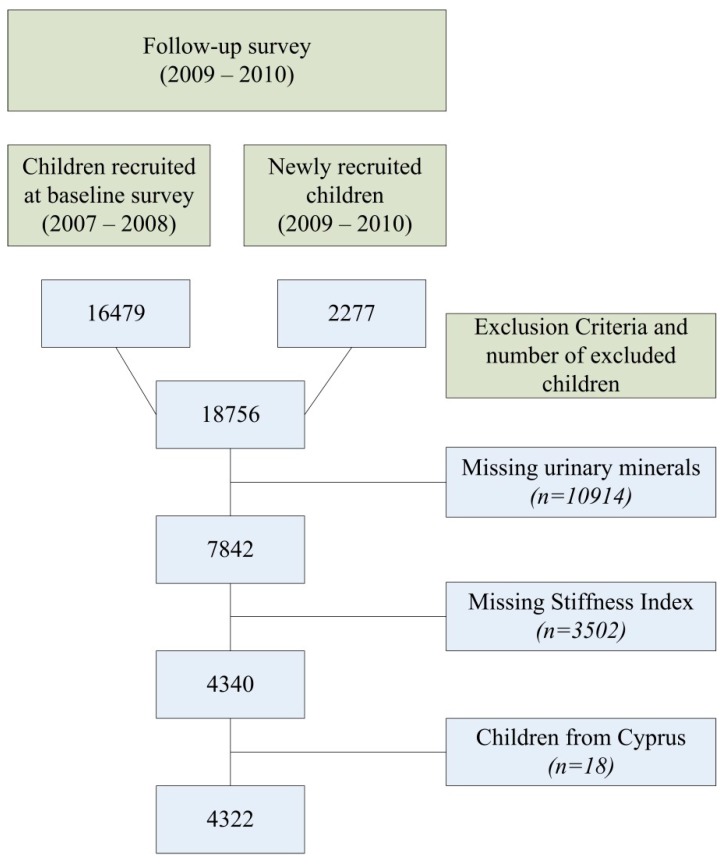
Number of included and excluded children per exclusion criteria.

This cross-sectional analysis is based on the Identification and Prevention of Dietary- and Lifestyle-Induced Health Effects in Children and Infants (IDEFICS) study (www.idefics.eu), a population-based multi-stage intervention cohort study investigating factors that influence the health, growth, and development of European children, with emphasis on obesity and its co-morbid conditions. Research goals and instruments have been described in detail [24,25]. The IDEFICS databases have not been released [26]. The study was conducted in eight European countries (Belgium, Cyprus, Estonia, Germany, Hungary, Italy, Spain and Sweden) and included two measurement periods: a baseline survey in 2007–2008 and a follow-up survey in 2009–2010 in both an intervention and a control region. The study population and drop-outs in the IDEFICS intervention and control regions at both baseline and follow-up survey, and the results of the intervention have been described elsewhere [27,28]. In this study, we only considered children who participated in the follow-up survey, recruited at baseline survey in 2007–2008 or newly recruited in 2009–2010 from both the control and intervention region, and with data on calcaneal QUS combined with available data on the five urinary mineral concentrations. Figure 1 summarises all exclusion criteria as well as the number of included and excluded children for this analysis. 

Data were complete for 4322 children (age 8.15 ± 1.83 years, range 3.1–11.9 years, 50.6% boys) from seven participating countries. Only 18 children from Cyprus were eligible and therefore all participants from Cyprus were excluded. We looked at differences in terms of urinary mineral concentrations between the group with and without available data on bone measurements. The 4340 children with available data on SI (age 8.15 ± 1.83 years, range 3.10–11.9 years, 50.5% boys, uK/Cr 3.64 ± 0.56, uPO_4_/Cr 0.04 ± 0.35) differed with respect to uK/Cr (*p* = 0.002), uPO_4_/Cr (*p* < 0.001) and age (*p* < 0.001) from the 3502 children without available bone measurements (age 7.78 ± 1.85 years, range 3.10–11.8 years, 49.1% boys, uK/Cr 3.68 ± 0.57, uPO_4_/Cr 0.07 ± 0.36). These significant findings could possibly be explained due to the age difference. The study was conducted according to the guidelines laid down in the Declaration of Helsinki and all procedures involving human subjects were approved by the ethical committee of each survey centre. Written informed consent was obtained prior to participation in the study from the parents of all participating children who gave oral consent.

### 2.2. Assessment of Calcaneal Bone Parameters

Calcaneal QUS (Lunar Achilles Insight, GE Healthcare, Milwaukee, WI, USA) was performed in all participants to assess speed of sound (SOS), broadband ultrasound attenuation (BUA) and bone stiffness index (SI). SOS, a measure of the ultrasound velocity inside the bone, describes the stiffness of a material by the ratio of the traversed distance to the transit time, in meters per second. BUA reflects the absorption of sound waves and is expressed as decibels per megahertz. SOS is in comparison to BUA a predictor of mechanical properties and therefore of bone strength in trabecular bone [29,30]. SI is calculated by a linear combination of BUA and SOS as (0.67 × BUA) + (0.28 × SOS) − 420 [31,32]. A detailed description is available [2,33]. Due to different registration settings of the QUS devices, BUA and SOS values were not available for all participants (BUA: *n* = 2258, SOS; *n* = 2263, and SI: *n* = 4340). A comparability study of five QUS devices used within the IDEFICS study was conducted on 91 subjects performing three repeated measurements per foot and device, revealing a significant deviation of the SI-values between the devices varying within a range of 0 to 5 SI units in average. Multiple multilevel regression analysis was used to control for the cluster design and considering that in each country a different device was used.

### 2.3. Assessment of Urinary Mineral Concentrations

Each child was provided with a urine container and detailed instruction sheet for the parents. On the morning of the examination day, the child needed to collect the first morning urine and bring it to school without cooling. Pre-analytical sample processing of urine samples was done at the local survey centers or at local laboratories. All urine samples were transported in cooling boxes, stored at −20 °C and shipped on dry ice to the central laboratory [25]. First morning urine samples were analysed centrally in an International Organization for Standardization 15,189 accredited laboratory. All urinary mineral concentrations were determined by photometric assay using Roche Integra 800 (Roche, Mannheim, Germany). Urinary concentrations were expressed in millimole per litre, except for creatinine (uCr) and uPO_4_ (gram per litre). Samples above the following threshold, according to the distribution, were excluded: 5 g/L for uCr, 500 mmol/L for uNa, 6 g/L for uPO_4_, 35 mmol/L for uMg, 250 mmol/L for uK and 20 mmol/L for uCa. This resulted in an exclusion of 949 participants for uCr, uMg and uCa on the one hand and an exclusion of 19 subjects after checking for extreme values on the other hand. All five urinary concentrations were standardised for uCr excretion by using ratios of uCr to the urinary mineral: uNa/Cr (mmol/g), uK/Cr (mmol/g), uMg/Cr (mmol/g), uPO_4_/Cr and uCa/Cr (mmol/g). In the text we refer to these standardized ratios with the term urinary mineral concentrations.

### 2.4. Assessment of Body Composition

A detailed description of the anthropometric measurements adopted in the IDEFICS study, including intra- and inter-observer reliability, is available [34]. In short, anthropometric measurements were performed in each survey center by trained researchers. Height was measured with a standard clinical Seca 224 stadiometer (Seca, Hamburg, Germany) to the nearest 0.1 cm. Weight was determined with a standard balance (BC 420 SMA; Tanita, Amsterdam, The Netherlands) to the nearest 0.1 kg, without shoes and in light clothing. The Tanita balance (adapted to the small foot size of children) also measured leg-to-leg impedance (ohm). The Tyrrell formula was used to calculate the fat-free mass (FFM, in kilograms) based on this impedance value [35]. Body mass index (BMI) was computed according to the following formula: BMI = weight (kg)/height^2^ (m^2^) [36]. For each child, *z*-scores of BMI were determined using the British reference population [37]. 

### 2.5. Questionnaire

A self-administered parental questionnaire was used to obtain information on the following variables: sex of the child and date of birth. The age of the child at time of examination was calculated using date of birth and date of examination.

### 2.6. Statistical Analysis

Descriptive data stratified by sex were examined with independent samples *t*-tests. Boys and girls were analysed together since there was no interaction observed between sex and bone variables (*p* > 0.05). One-Way ANOVA comparison of means and *post hoc* Bonferroni tests were used to examine the differences between countries in terms of urinary mineral concentrations, age and BMI *z*-score. To examine, in the above parameters, the proportion of the variance explained by differences between countries, the effect size (eta^2^, η^2^) was calculated and interpreted according to the categories: η^2^ = 0.02 is considered small, η^2^ = 0.13 is considered medium and η^2^ = 0.26 is considered large [38]. To show differences in urinary mineral concentrations between countries, bar charts of means with error bars of standard error (SE) were used (Figure S1). When SE error bars overlap, the difference between mean concentrations is not statistically significant (*p* > 0.05). For the following analyses, natural logarithmic transformation of all urinary mineral concentrations was done in order to achieve a satisfactory pattern (normal distribution) of all residuals. First, Pearson correlation coefficients were performed to assess associations between the various urinary mineral concentrations among each other. Cohen’s conventions on effects were used to identify small, medium or large effects for the Pearson correlation coefficients (respectively 0.1, 0.3 and 0.5) [39]. Second, two-step clustering based on likelihood measures was applied to identify patterns in urinary excretion [40]. Ward’s hierarchical clustering method was used to identify distinctive homogenous clusters based on uNa, uK, uPO_4_, uMg and uCa. After having identified the optimal number of clusters, the clustering was fine-tuned by using the non-hierarchical K-means clustering method. One-Way ANOVA comparison of means and *post hoc* Bonferroni tests were used to profile the clusters in terms of QUS parameters (SOS, BUA and SI), age and BMI z-score. Chi^2^ tests were used to profile the clusters in terms of sex and country. Finally, multiple multilevel regression analysis using linear mixed models were conducted taking into account the clustering of children within countries. These analyses were used to study the association between each urinary mineral concentration separately as an independent variable and the dependent variables BUA, SOS, and SI. The variables age, sex and FFM were included as confounders in all analysis, since they are associated with calcaneal QUS parameters. In a second model, uNa was added as confounder in the analysis assessing the association between uCa and the calcaneal QUS parameters. The unstandardized regression coefficients are given for one unit increase on log-scale. Moreover, the coefficient of the significant associations were calculated for an urinary mineral that would multiply tenfold, using the formula β’(tenfold increase) = β × ln(10). All statistical analyses were performed using the PASW Statistics Program, version 22.0.0 (SPSS, Inc., Chicago, IL, USA); statistical results with *p* < 0.05 were considered statistically significant. 

## 3. Results

### 3.1. Subject Characteristics

The mean and standard deviation of age, anthropometric variables, calcaneal QUS parameters and urinary mineral concentrations are summarized in Table 1. The study sample consisted of 4322 children (50.6% boys). The mean FFM, uCr, uK and uPO_4_ were slightly higher in boys compared to girls (*p* < 0.05). No sex differences in QUS parameters were found. 

### 3.2. Differences in Urinary Mineral Concentrations and Calcaneal QUS Parameters Between Countries

The mean and standard deviation of urinary mineral concentrations, calcaneal QUS parameters, age, sex, BMI *z*-score and FFM per country are summarized in Table 2. Only sex was not significant different (*p* = 0.625) between countries. Figure S1 shows the mean concentrations and SE of uCr and urinary mineral concentrations after correction for uCr (A–F) in the different countries. For example, all urinary mineral concentrations differed statistically between Estonia and Spain. All urinary mineral concentrations were corrected for uCr and significantly different (*p* < 0.001) between countries. Only a small effect of differences in countries on all urinary mineral concentrations was observed (η^2^ = 0.03–0.08). 

### 3.3. Associations between the Concentrations of the Various Urinary Mineral Concentrations

Correlation analyses between the concentrations of the various urinary mineral concentrations are presented in Table 3. Significant positive associations with medium effect size were found between uNa/Cr and (1) uCa/Cr and (2) uK/Cr. Positive associations with medium effects were found between uPO_4_/Cr and (1) uCa/Cr and (2) uMg/Cr. A positive association with an almost large effect size was found between uCa/Cr and uMg/Cr. Significant positive associations with small effect size were found between uNa/Cr and (1) uMg/Cr and (2) uPO_4_/Cr. Significant negative associations with small effect size were found between uK/Cr and (1) uCa/Cr and (2) uMg/Cr but not with (3) uPO_4_/Cr.

### 3.4. Patterns in Concentrations of Urinary Mineral Concentrations

Cluster analysis resulted in a three-cluster solution as the most appropriate to identify patterns in urinary mineral concentrations (after correcting for creatinine). Descriptive statistics of the clusters and the associations between the identified clusters and several parameters are shown in Table 4. Cluster 1 (C1) accounted for 32.9% (*n* = 1409) of the sample. C1 was characterized by low uCa/Cr, uNa/Cr, uMg/Cr and uPO_4_/Cr concentrations, and an average uK/Cr concentration. Cluster 2 (C2) accounted for 40.7% (*n* = 1742) of the sample and was characterized by medium concentrations of uCa/Cr, uNa/Cr, uMg/Cr and uPO_4_/Cr, and a low concentration of uK/Cr. Cluster 3 (C3) accounted for 26.4% (*n* = 1171) of the sample. C3 was characterized by high concentrations of all five urinary mineral concentrations. 

One-Way ANOVA analyses showed that the SOS of children within C3 was significantly higher than in C1 and C2. The same was observed for the mean of SI between the children within C3 and C2. The mean of BUA was significantly lower in C3 compared to C1 and C2. The youngest children were in C3 and the oldest in C1. The average BMI z-score is significantly lower in C3 (0.36 ± 1.25) compared to C1 (0.50 ± 1.22) and C2 with the highest BMI (0.56 ± 1.24). Chi^2^ tests showed that uCr excretions differed significantly among the clusters with the highest in C1 and the lowest in C3. A significant difference (Pearson chi^2^ = 0.026) was found regarding to the distribution of sex. The presence of the various countries is significant different between the clusters (Pearson chi^2^ < 0.001).

### 3.5. Associations between Concentrations of Urinary Mineral Concentrations and Calcaneal QUS Parameters

Multiple multilevel regression analyses indicated that the cluster random sampling design of the study (using different countries) did have an influence on the QUS parameters (*p* < 0.001, results no shown). Table 5 shows the results of the multiple regression analyses investigating the independent relationship of urinary mineral concentrations and calcaneal QUS parameters when controlling for age, sex and FFM. All confounders showed a significant association with the outcome variables (results not shown). uK/Cr was positively associated with SOS (B = 3.086; *p* = 0.027; *R*^2^ = 0.196). uNa/Cr was positively associated with BUA (B = 1.315; *p* = 0.013; *R*^2^ = 0.369) and SI (B = 1.324; *p* < 0.001; *R*^2^ = 0.034). In a second model uNa/Cr was added as a covariate whereby a significant negative association between uCa/Cr and SI was found, however the *R*^2^ of the model was very low (B = −0.542; *p* = 0.03; *R*^2^ = 0.035). Adjusted coefficients were calculated for the significant associations. When the urinary mineral multiplies tenfold, than SOS increases with 7.106 (uK/Cr), BUA with 3.028 (uNa/Cr) and SI with 3.049 (uNa/Cr) and −1.248 (uCa/Cr). No other significant associations between urinary mineral concentrations and calcaneal QUS parameters were found.

## 4. Discussion

This cross-sectional study found differences in urinary mineral concentrations adjusted for creatinine concentration and calcaneal QUS parameters in 4322 pre-adolescent children across seven European countries. Additionally, the associations between urinary mineral concentrations and calcaneal QUS parameters were assessed.

### 4.1. Urinary Mineral Concentrations in Different Countries

Referring to the first hypothesis, significant differences of urinary mineral concentrations between countries were observed. In terms of uCr excretions, Spain has the lowest and Estonia the highest levels. Generally, creatinine, the metabolite of creatine phosphate in muscle, is used to correct for variable dilutions among spot samples [41]. Estonian children were significantly older and therefore had a higher FFM, which could explain the highest uCr [42]. Our results suggest a variability in the dilution of the urine sample, with Spanish children having the most diluted morning urine spot samples. This difference in uCr between countries must be kept in mind when considering the differences in the urinary mineral concentrations between these countries after correction for uCr (e.g., highest level of uNa/Cr in Spain compared to lowest levels in Estonia). In contrast to our country differences, considering the mean value (95%CI) of uCr excretion from all seven countries (104.2 mg/dL (102.90–105.49)), our findings are similar to the mean uCr from 3078 6–11 year old children from the NHANES III study (102.1 mg/dL (98.91–105.2)) [41]. 

Next, the differences in the urinary mineral concentrations can also be partly explained by the difference in dietary patterns [43]. Huybrechts *et al.* [44] found in the same IDEFICS-population large differences between dairy consumption frequencies among the countries with the highest consumption in Cyprus and Spain, and the lowest in Hungary, Belgium and Italy. Additionally, the researchers observed in Belgian children with the lowest dairy product consumption, the lowest uCa/Cr and the highest uK/Cr [44]. An association between a diet rich in fruit and vegetables and uK concentrations has been found in a study on Japanese adults [45]. In contrast with these findings, no associations were found between uK excretion and intakes of fruit and vegetables in a 5-day randomized controlled study of 12 men in Denmark [46] as well as in a study with the IDEFICS population—being the same population as the one in this study [44]. 

Since assessment of diet is likely to underestimate the Na intake, uNa is a good marker for the intake of Na [47]. Spain and Hungary have the highest levels of uNa/Cr, indicating a more Na rich diet and linked to processed food [11], and Estonia the lowest levels of uNa/Cr. In case of uPO_4_ the association with dietary intake is more difficult to make since its homeostasis is determined PO_4_ modulation of intestinal uptake of dietary PO_4_, renal PO_4_ reabsorption and excretion, and the exchange of PO_4_ between extracellular and bone storage pools, involving multiple regulators. In states of metabolic PO_4_ equilibrium and normal renal function, the amount of PO_4_ appearing in the urine can serve as a rough approximation of the amount absorbed in the intestine, but during periods of rapid growth and development, as in infancy and puberty, a positive PO_4_ balance is established [12]. In accordance with PO_4_, dietary intake of Mg is not well associated with uMg excretions. The kidney has a very large capacity for Mg excretion and when the renal threshold is exceeded, most of the excess filtered Mg is excreted into the urine [48]. Under basal conditions the small intestine absorbs 30%–50% of Mg intake, but this percentage diminishes with increasing amount of Mg intake [48]. In general Mg is at risk for being deficient in the diet [49]. Consumption of a rather plant-based fruit and vegetable-rich diet results in higher intakes of mineral cations, especially of K and Mg [50].

Clustering the urinary mineral excretions revealed three main groups: low, middle and high levels of urinary mineral concentrations. Country was a contributing factor but had only a small effect on the variation in the urinary mineral concentrations. In accordance with above results, Spanish children were mostly represented in the group of high levels of urinary mineral concentrations; Estonian children almost equally represented in the groups with low and middle levels of urinary mineral concentrations. Probably, this can be partly explained by the average low and high uCr levels found in the Spanish and Estonian children, respectively. Apart from a difference in countries between clusters, a small difference between the clusters was also found for age, sex and BMI *z*-score. The higher concentrations of urinary mineral concentrations can possibly be explained by a difference in dietary pattern, in particular a diet more rich in dairy products, fruit and vegetables in children [51,52]. Subsequently, this could also partly be explained by the lower levels of uCr in this younger age groups, resulting in higher urinary mineral concentrations. The BMI *z*-score is the lowest in the group with high levels of urinary excretions, suggesting a difference in dietary patterns.

Referring to our second hypothesis, the positive association between uNa/Cr and uCa/Cr points towards the physiological pathway that, on renal level, the presence of Na prevents the reabsorption of Ca [15]. In line with our findings, previous studies reported a positive association between uNa and uCa in American girls 8 to 13 years of age [20], in German children and adolescents aged 6 to 18 years [21], in Puerto Rican girls aged 11 to 15 years [19], in 77 white American children aged 4 to 17 years [53] and in 154 Australian and 32 Finnish adults [54,55]. However, Safarinejad [56] found no relationship between uCa/Cr and uNa/Cr in 900 Iranian children. Referring to our third hypothesis, the inverse association between uK/Cr and uCa/Cr points towards the physiological pathway that, on renal level, the presence of K prevents the excretion of Ca. Osorio and Alon [53] found similar results in 100 white and black American children aged 4 to 17 years. Third, a negative association between uK and uMg was found. In the study of Jones *et al.* [57] with 136 boys aged 16, also an inverse association (*r* = −0.24, *p* < 0.05) between uK and uMg measured from first morning spot samples was found. They stated that this might be a potential unexplored mechanism of action [57]. Fourth, we found a positive association between uMg/Cr and uCa/Cr, and between uMg/Cr and uNa/Cr. The metabolism of Mg is not yet clear, Palacios *et al.* [19] reported that physiological control mechanisms for Ca may be shared with Mg. Ca could inhibit enteral Mg absorption and vice versa, but this has been contradicted as well [11]. Finally, we found that uPO_4_/Cr was positively correlated with uCa/Cr, uNa/Cr and uMg/Cr. Not much literature is found on the relationship between uPO_4_ and uCa, uNa and uMg. It is known that Ca and P are both essential nutrients for bone and are closely related to parathyroid hormone (PTH), one of the most important regulators of bone metabolism. PTH induces pathways leading to increased uPO_4_ excretion and decreased uCa excretion [58], which does not articulate well with the positive association between uCa/Cr and uPO_4_/Cr found in this study. A possible explanation could be diet whereby some foods rich in P are also good sources of Ca (e.g., milk products).

### 4.2. Urine and QUS Results

The results pertaining to the second goal revealed differences in bone health according to low, middle or high amount of minerals in urine. Children with high concentrations of urinary mineral concentrations had a significantly higher SOS and SI but a lower BUA. Further, differences between the parameters SOS and BUA were observed and can point to different physiological pathways. Ultrasound velocity inside the bone (measured by SOS), more than ultrasound attenuation through bone (measured by BUA), measures the mechanical properties of bone [29]. This may indicate that a diet rich in minerals may lead to a better microarchitecture of bone, and thus a better SOS. Based on multiple multilevel regression analysis, we found a positive association between uK and SOS. This could mean that a healthy diet (rich in K) is positively associated with bone parameters presumably via its protective role in the retention of Ca. Jones *et al.* [18] found similar results between uK, from a single, non-fasting urine sample, and bone mineral density (BMD) measured *via* DEXA in 330 children being 8 year olds. However, Tylavsky *et al.* [23] found no relationship between the intake of fruit and vegetables and whole body DXA in 65 girls. Referring to our fourth hypothesis, a rather surprisingly positive association of uNa with BUA and SI was found. This contradicts previous study results that found a non-beneficial association between uNa and bone turnover markers [54,57] or a non-existent association between uNa and BMD measured via DEXA at multiple sites [18]. This association could be explained by the difference in bone measurement methods—bone turnover markers or DEXA (at different body sites) compared to QUS–, or different methods of urine collection (24h urine *vs.* single voids). No association was found between uCa/Cr and bone, but after correction for uNa/Cr, uCa/Cr was negatively associated with SI. This could mean that more uCa loss results in lower calcaneal QUS parameters (SI). This is in line with existing literature. Matkovic *et al.* [20] found an inverse association between uCa and BMD and bone mineral content (BMC) (measured via DEXA of the total body and forearm). Shi *et al.* [21] found a negative association between uCa and volumetric BMD and bone mineral content (measured *via* pQCT of the forearm) but not with the cross-sectional area and Strength Strain Index.

### 4.3. Strengths and Limitations

The availability of a large population-based sample of children 3 to 12 years of age from seven European countries is a strength of this study. The analyses could be corrected for potential confounders, since the availability of a large battery of measurements. Moreover, strict standardisation procedures were followed during the data collection [25,59,60]. The fact that all urine samples collected in this survey were all analysed in the same laboratory using the same standard protocol and materials, reduced analytical variations that might have affected the results obtained. An important limitation is the use of only one first morning urine sample instead of a 24-h urine collection whereby the excretion of the urinary mineral concentrations could be underestimated or overestimated. A longitudinal approach collecting multiple samples or 24-h collections may be required to assess more accurately the habitual mineral excretions [61,62]. Because of the use of morning urine samples, the urinary mineral concentrations were standardised for uCr excretions by calculating the ratios of uCa, uNa, uK, uMg and uPO_4_ to uCr concentrations. The uncertainty of uCr standardisation is increased when studying single voids rather than 24-h urine samples [63]. However, seen the magnitude of the study, the use of spot urine samples considerably increased the logistical feasibility and the convenience for the study participants, resulting in a high number of collected samples and therefore an increased statistical power. Furthermore, the influence of body size on uCr excretions was overcome by the correction for sex, age and FFM in multiple regression analysis [64]. In addition, it is notable to mention that according to the protocol instructions the urine samples were not necessarily fasted samples. A second limitation is the cross-sectional nature of this study, for this analysis, no longitudinal data was available. A third limitation is the lack of a dietary record of the day before the urine sample collection. As a result, no association could be studied between the dietary intake and the excretion of urinary mineral concentrations. Moreover, it was not possible to comment on the adequacy of the daily intakes of the studied minerals compared to the recommendations. Fourthly, for future analysis it would be of interest to look at young and older age groups, and other confounding factors (e.g., physical activity and dietary pattern). Due to the explorative character of the study and not to reduce the statistical power, we decided to analyse all children but with adjustment for age. Fifthly, pubertal stages were not assessed. Some of the older children may have entered puberty at the time of their examination.

Finally a few remarks must be made regarding the bone measurements with the QUS method. First, some inaccuracy in the measurements can occur due to difficult positioning and immobilisation of the small feet in children. Therefore trained and only a limited number of researchers were used to obtain the QUS data. Second, intra-device differences were found in a small reliability study which could mean a systematic difference between countries. Therefore multiple multilevel regression analysis was used to control for the cluster design. Third, the BUA and SOS values measured by the QUS were not registered for all participants, due to different registration settings of the measuring device. Finally the QUS method is a practical device, but is not yet accepted as a standard measurement method in children [65,66]. Nevertheless, using the portable and radiation-free QUS device could increase the participation ratio in a pediatric population which is necessary in this kind of research in large population based cohorts (e.g., field work can be done at schools).

## 5. Conclusions

In conclusion, this study points towards the importance of a good mineral balance, even in healthy children. Inadequate levels of minerals in the body during this critical period of attaining maximal peak bone mass may have negative implications on bone formation, and could even lead to osteoporosis later in life. These findings should be confirmed in longitudinal studies that include the food intake and repeated measurement of urinary mineral concentrations to better estimate usual intake and minimize bias.

## Figures and Tables

**Table 1:** Descriptive characteristics of the studied children by gender. Mean and standard deviation (SD).

Characteristic	Boys		Girls		Total		*p*-Value ^a,b^ Gender Difference
	*n* = 2187		*n* = 2135		*n* = 4322		
	Mean	SD	Mean	SD	Mean	SD	
Calcaneal SI	82.95	14.20	82.77	14.26	82.86	14.23	0.675
Calcaneal SOS ^c^ (m/s)	1590.10	43.83	1589.90	44.09	1590.00	43.94	0.914
Calcaneal BUA ^d^ (dB/MHz)	87.97	16.96	86.71	16.10	87.36	16.55	0.070
Age (years)	8.11	1.83	8.19	1.83	8.15	1.83	0.155
Height (cm)	130.86	11.91	130.62	12.24	130.74	12.07	0.526
Weight (kg)	30.64	9.98	30.44	9.85	30.54	9.90	0.509
Fat-free mass (kg)	21.44	5.06	19.90	5.13	20.67	5.15	**<0.001**
uCr (g/L)	1.06	0.44	1.03	0.43	1.04	0.43	**0.014**
uCa (mmol/L)	3.03	2.22	3.00	2.25	3.02	2.23	0.734
uCa/Cr (mmol/g)	3.11	2.27	3.15	2.30	3.13	2.29	0.501
uNa (mmol/L)	138.86	54.44	133.13	53.91	136.03	54.25	**0.001**
uNa/Cr (mmol/g)	151.69	84.22	150.67	87.07	151.18	85.63	0.696
uK (mmol/L)	43.46	23.04	38.86	20.84	41.19	22.10	**<0.001**
uK/Cr (mmol/g)	47.32	34.33	43.69	31.76	45.53	33.13	**<0.001**
uMg (mmol/L)	6.06	2.71	6.02	2.85	6.04	2.78	0.637
uMg/Cr (mmol/g)	6.01	2.15	6.08	2.16	6.04	2.16	0.238
uPO_4_ (g/L)	1.14	0.49	1.08	0.49	1.11	0.49	**<0.001**
uPO_4_/Cr	1.12	0.36	1.09	0.35	1.10	0.36	**0.004**

SI, stiffness index. SOS, speed of sound. BUA, broadband ultrasound attenuation. uCr, urinary creatinine. uCa, urinary calcium. uCa/Cr, urinary calcium/creatinine ratio. uNa, urinary sodium. uNa/Cr, urinary sodium/creatinine ratio. uK, urinary potassium. uK/Cr, urinary potassium/creatinine ratio. uMg, urinary magnesium. uMg/Cr, urinary magnesium/creatinine ratio. uPO_4_, urinary phosphate. uPO_4_/Cr, urinary phosphate/creatinine ratio. **^a^** Independent samples *t*-tests. **^b^** *p*-values < 0.05 are indicated in bold. ^c^ *n* = 1163 for boys, *n* = 1098 for girls and total *n* = 2261. ^d^ *n* = 1159 for boys, *n* = 1097 for girls and total *n* = 2256.

**Table 2:** Descriptive characteristics of the studied children, comparison between the countries. Mean and standard deviation (SD).

Characteristic	Belgium		Estonia		Germany		Hungary		Italy		Spain		Sweden		*p*-Value ^b^	Eta Squared
	*n* = 542 ^a^		*n* = 271		*n* = 951		*n* = 803		*n* = 1190		*n* = 200		*n* = 365			
	Mean	SD	Mean	SD	Mean	SD	Mean	SD	Mean	SD	Mean	SD	Mean	SD		
uCa/Cr (mmol/g)	2.71	2.02	2.38	1.57	2.81	2.12	3.40	2.29	3.28	2.44	4.08	2.53	3.56	2.47	**<0.001**	0.03
uNa/Cr (mmol/g)	143.14	78.68	125.68	77.70	148.58	85.77	171.93	95.53	142.03	79.59	204.12	91.95	144.04	70.09	**<0.000**	0.04
uK/Cr (mmol/g)	56.60	38.18	39.69	28.23	46.70	34.73	41.50	32.09	41.12	27.84	55.74	40.49	48.22	32.37	**<0.001**	0.03
uMg/Cr (mmol/g)	5.86	2.18	4.82	1.59	5.83	2.06	6.60	2.24	6.07	2.09	6.60	2.25	6.17	2.23	**<0.001**	0.04
uPO_4_/Cr (g/L)	1.11	0.35	1.01	0.31	1.08	0.34	0.99	0.34	1.10	0.33	1.41	0.40	1.27	0.38	**<0.001**	0.08
uCr (g/L)	1.03	0.40	1.26	0.51	1.08	0.44	0.98	0.46	1.05	0.41	0.84	0.32	1.03	0.39	**<0.001**	0.03
SI	93.67	15.66	81.25	10.04	79.68	12.24	88.51	14.63	76.13	11.59	86.11	10.29	83.99	13.33	**<0.001**	0.18
SOS (m/s)	1623.27	49.47	1582.93	20.61	1590.2	39.36	1609.24	57.42	1567.36	32.68	1589.97	28.03	/	/	**<0.000**	0.19
	*n* = 349		*n* = 261		*n* = 522		*n* = 272		*n* = 667		*n* = 190		*n* = 0			
BUA (dB/MHz)	90.14	24.61	87.03	10.76	84.55	15.46	87.09	17.22	86.91	14.17	92.32	12.35	/	/	**<0.001**	0.02
	*n* = 347		*n* = 261		*n* = 519		*n* = 272		*n* = 667		*n* = 190		*n* = 0			
Age (years)	7.69	1.55	9.65	1.06	8.14	1.82	7.94	2.06	8.15	1.80	8.19	1.35	8.17	1.92	**<0.001**	0.05
BMI z-score	0.01	0.92	0.28	1.05	0.26	1.04	0.14	1.34	1.27	1.21	0.65	1.06	0.13	1.00	**<0.001**	0.16
FFM (kg)	18.87	4.26	24.53	4.17	20.43	4.91	20.43	5.51	21.08	5.38	20.41	5.29	20.44	4.89	**<0.001**	0.05
	*n* = 539		*n* = 271		*n* = 950		*n* = 803		*n* = 1170		*n* = 199		*n* = 365			
% boys	51.7		47.6		51.2		48.6		52.2		51		48.8		**0.625** ^c^	

uCa/Cr, urinary calcium/creatinine ratio. uNa/Cr, urinary sodium/creatinine ratio. uK/Cr, urinary potassium/creatinine ratio. uMg/Cr, urinary magnesium/creatinine ratio. uPO_4_/Cr, urinary phosphate/creatinine ratio. uCr, urinary creatinine. SI, stiffness index. SOS, speed of sound. BUA, broadband ultrasound attenuation. BMI *z*-score, *z*-score of Body Mass Index. FFM, fat-free mass. ^a^ number of participants per country, unless indicated otherwise in the table; ^b^ One-Way ANOVA; ^c^ Pearson chi^2^.

**Table 3:** Results of Pearson’s correlations between urinary mineral concentrations (*n* = 4322).

Urinary Mineral Concentrations	uNa/Cr ^a^	uK/Cr	uMg/Cr	uPO_4_/Cr
uCa/Cr	0.328 *	−0.116 *	0.495 *	0.343 *
uNa/Cr		0.414 *	0.199 *	0.162 *
uK/Cr			−0.061 *	−0.005
uMg/Cr				0.465 *

Abbreviations as in Table 2. **^a^** all urinary mineral concentrations were natural log transformed. * *p*-value < 0.001.

**Table 4:** Descriptive characteristics of the clusters and One-Way ANOVA analysis urinary mineral concentrations and bone health. Mean and standard deviation (SD).

Characteristic	Cluster 1 ^c^		Cluster 2		Cluster 3		Eta Squared
	*n* = 1409 ^d^		*n* = 1742		*n* = 1171		
	Mean	SD	Mean	SD	Mean	SD	
uCa/Cr (mmol/g)	1.03	0.46	4.04	1.92	4.30	2.36	0.41
uNa/Cr (mmol/g)	111.80	54.83	123.68	45.74	239.49	97.74	0.40
uK/Cr (mmol/g)	45.52	28.87	27.92	9.66	71.73	41.99	0.28
uMg/Cr (mmol/g)	4.75	1.62	6.45	1.89	7.00	2.36	0.18
uPO_4_/Cr	0.96	0.31	1.12	0.30	1.25	0.42	0.10
SI	82.99	13.80	82.08 ^a^	13.82	83.86 ^a^	15.25	0.003
SOS (m/s)	1587.72 ^a^	39.59	1584.24 ^b^	40.25	1601.49 ^a,b^	51.90	0.03
	*n* = 799		*n* = 868		*n* = 594		
BUA (dB/MHz)	88.41 ^a^	15.39	87.92 ^b^	15.73	85.11 ^a,b^	18.89	0.007
	*n* = 796		*n* = 867		*n* = 593		
Age (years)	8.41 ^a^	1.74	8.38 ^b^	1.76	7.50 ^a,b^	1.88	0.05
BMI *z*-score	0.50 ^a^	1.22	0.56 ^b^	1.24	0.36 ^a,b^	1.25	0.004
FFM (kg)	21.36 ^a^	5.01	21.17 ^b^	4.94	19.11 ^a,b^	5.29	0.03
	*n* = 1400		*n* = 1734		*n* = 1163		
Cr (g/L)	1.19 ^a^	0.47	1.10 ^a^	0.38	0.72 ^a^	0.26	0.21
% boys ^e^	51.5		48.2		53.1		
Country ^f^	32.8% Italy 40.6% Estonia 40.8% Belgium 29.3% Sweden 38.1% Germany 23.8% Hungary 14.0% Spain		45.9% Italy 44.3% Estonia 28.6% Belgium 36.4% Sweden 37.5% Germany 45.8% Hungary 31.5% Spain		21.3% Italy 15.1% Estonia 30.6% Belgium 34.2% Sweden 24.4% Germany 30.4% Hungary 54.5% Spain		

uCa/Cr, urinary calcium/creatinine ratio. uNa/Cr, urinary Sodium/creatinine ratio. uK/Cr, urinary Potassium/creatinine ratio. uMg/Cr, urinary Magnesium/creatinine ratio. uPO_4_/Cr, urinary Phosphate/creatinine ratio. SI, stiffness index. SOS, speed of sound. BUA, broadband ultrasound attenuation. BMI, Body Mass Index. FFM, fat-free mass. uCr, urinary creatinine. ^a,b^ Mean groups within a column with similar superscript letters were significantly different (P<0.05). **^c^** all urinary mineral concentrations were natural log transformed for clustering. **^d^** Number of participants per group, unless indicated otherwise in the table. ^e^ Pearson chi^2^ = 7.326; *p* = 0.026. ^f^ Pearson chi^2^ = 207.910; *p* < 0.001, percentages should be read horizontally.

**Table 5:** Results of multiple regression analysis (mixed models) controlled for cluster design.

Urinary Mineral Concentrations ^c^	SOS			BUA			SI		
	*n* = 2261			*n* = 2256			*n* = 4322		
	B (95%CI)	*p-*Value ^d^	*R*^2^	B (95%CI)	*p*-Value	*R*^2^	B (95%CI)	*p*-Value	*R*^2^
uCa/Cr ^a^	−0.356 (−2.112;1.399)	0.691	0.195	0.280 (−0.370;0.929)	0.389	0.367	−0.234 (−0.699;0.232)	0.325	0.031
uNa/Cr ^a^	2.496 (−0.301;5.294)	0.080	0.196	1.315 (0.281;2.348)	**0.013**	0.369	1.324 (0.599;2.049)	**<0.001**	0.034
uK/Cr ^a^	3.086 (0.348;5.824)	**0.027**	0.197	0.614 (−0.399;1.626)	0.235	0.368	0.453 (−0.261;1.167)	0.213	0.031
uMg/Cr ^a^	−0.429 (−4.258;3.400)	0.826	0.195	0.374 (−1.045;1.794)	0.605	0.367	−0.191 (−1.214;0.831)	0.713	0.031
uPO_4_/Cr ^a^	1.515 (−3.120;6.149)	0.522	0.195	1.630 (−0.082;3.341)	0.062	0.368	0.704 (−0.459;1.867)	0.236	0.031
uCa/Cr ^b^	−0.888 (−2.722;0.946)	0.342	0.197	0.044 (−0.634;0.722)	0.898	0.369	−0.542 (−1.029;−0.054)	**0.029**	0.035

uCa/Cr, urinary calcium/creatinine ratio. uNa/Cr, urinary Sodium/creatinine ratio. uK/Cr, urinary Potassium/creatinine ratio. uMg/Cr, urinary Magnesium/creatinine ratio. uPO_4_/Cr, urinary Phosphate/creatinine ratio. SI, stiffness index. SOS, speed of sound. BUA, broadband ultrasound attenuation. B, Unstandardized regression coefficients. *R*^2^, overall Adjusted R square. ^a^ Corrected for age, gender and fat-free mass. ^b^ Corrected for age, gender, fat-free mass and uNa/Cr. ^c^ all urinary mineral concentrations were natural log transformed. **^d^** *p*-values < 0.05 are indicated in bold.

## Supplementary Materials

The following are available online at www.mdpi.com/1660-4601/13/5/471/s1. Figure S1. Comparison of urinary biomarkers between countries using bar charts of means with error bars of standard error. (A) Calcium/Creatinine; (B) Sodium/Creatinine; (C) Potassium/Creatinine; (D) Magnesium/Creatinine; (E) Phosphate/Creatinine; (F) Creatinine.
