# Supplementary material for: Urinary Mineral Concentrations in European Pre-Adolescent Children and Their Association with Calcaneal Bone Quantitative Ultrasound Measurements
Source: Int J Environ Res Public Health. 2016 May 5;13(5):471. doi: 10.3390/ijerph13050471 (PMC4881096; doi:10.3390/ijerph13050471)
Supplement: Supplementary file 1 [file ijerph-13-00471-s001.pdf]

Supplementary Materials: Urinary Mineral Concentrations in European Pre-Adolescent Children and Their Association with Calcaneal Bone Quantitative Ultrasound Measurements

Karen Van den Bussche, Diana Herrmann, Stefaan De Henauw, Yiannis A. Kourides, Fabio Lauria, Staffan Marild, Dénes Molnár, Luis A. Moreno, Toomas Veidebaum, Wolfgang Ahrens and Isabelle Sioen on behalf of the IDEFICS Consortium

Figure S1 shows the mean concentrations and SE of urinary creatinine and electrolytes after correction for creatinine (A–F) in the different countries. When SE error bars overlap, the difference between mean concentrations is not statistically significant ($p > 0.05$). All urinary electrolytes were corrected for creatinine and significantly different ($p < 0.001$) between countries. Only a small effect of differences in countries on all urinary electrolytes was observed ($\eta^2 = 0.03–0.08$).

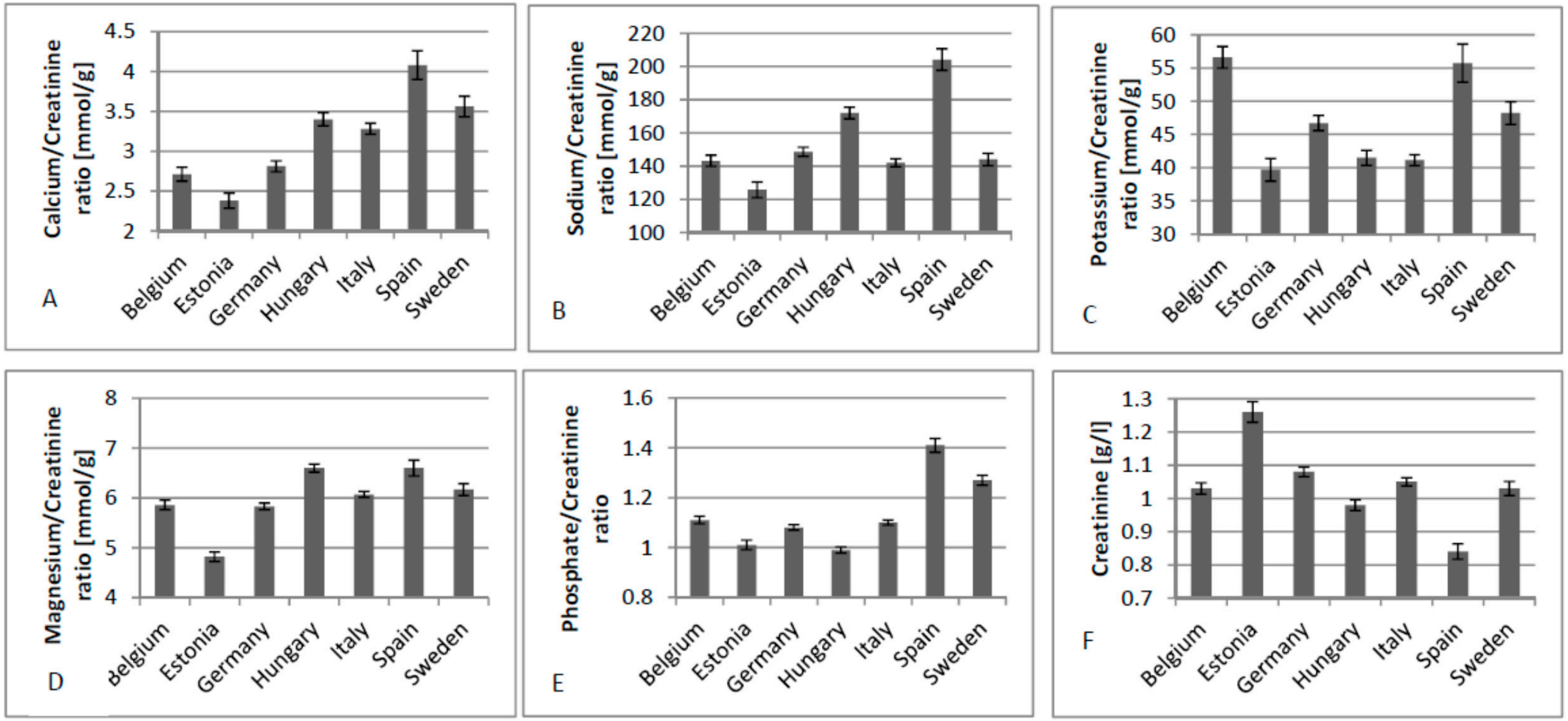

Number of participants	
Belgium	542
Estonia	271
Germany	951
Hungary	803
Italy	1190
Spain	200
Sweden	365
Total	4322

Figure S1. Comparison of urinary biomarkers between countries using bar charts of means with error bars of standard error. (**A**) Calcium/Creatinine; (**B**) Sodium/Creatinine; (**C**) Potassium/Creatinine; (**D**) Magnesium/Creatinine; (**E**) Phosphate/Creatinine; (**F**) Creatinine.

© 2016 by the authors; licensee MDPI, Basel, Switzerland. This article is an open access article distributed under the terms and conditions of the Creative Commons by Attribution (CC-BY) license (http://creativecommons.org/licenses/by/4.0/).
